# Low-cost peer-taught virtual research workshops for medical students in Pakistan: a creative, scalable, and sustainable solution for student research

**DOI:** 10.1186/s12909-021-02996-y

**Published:** 2021-11-01

**Authors:** Ronika Devi Ukrani, Ayesha Niaz Shaikh, Russell Seth Martins, Syeda Sadia Fatima, Hamna Amir Naseem, Mishall Ahmed Baig

**Affiliations:** 1 Medical College, Aga Khan University, Stadium Road, 74800 Karachi, Pakistan; 2 Research Mentor, Research and Development Wing, Society for Promoting Innovation in Education, Center for Innovation in Medical Education, Aga Khan University, Stadium Road, Karachi, 74800 Pakistan; 3 Department of Biological and Biomedical Sciences, Aga Khan University, Stadium Road, 74800 Karachi, Pakistan; 4 Medical College, DOW University of Health Sciences, Karachi, 74200 Pakistan

**Keywords:** Medical students, Online learning, Peer group, COVID-19

## Abstract

**Background:**

Pakistan has not been a major contributor to medical research, mainly because of the lack of learning opportunities to medical students. With the increase in online learning systems during COVID-19, research related skills can be taught to medical students via low-cost peer taught virtual research workshops.

**Aim of the Study:**

To assess the effectiveness of a comprehensive low-cost peer-taught virtual research workshops amongst medical students in Pakistan.

**Methods:**

This quasi-experimental study assessed the effectiveness of five virtual research workshops (RWs) in improving core research skills. RWs for medical students from across Pakistan were conducted over Zoom by medical students (peer-teachers) at the Aga Khan University, Pakistan, with minimal associated costs. The content of the workshops included types of research, ethical approval and research protocols, data collection and analysis, manuscript writing, and improving networking skills for research. Improvement was assessed via pre-and post-quizzes for each RW, self-efficacy scores across 16 domains, and feedback forms. Minimum criteria for completion of the RW series was attending at least 4/5 RWs and filling the post-RW series feedback form. A 6-month post-RW series follow-up survey was also emailed to the participants.

**Results:**

Four hundred medical students from 36 (/117; 30.8%) different medical colleges in Pakistan were enrolled in the RWs. However, only 307/400 (76.75%) medical students met the minimum requirement for completion of the RW series. 56.4% of the participants belonged to the pre-clinical years while the rest were currently to clinical years. The cohort demonstrated significant improvement in pre-and post-quiz scores for all 5 RWs (*p* <  0.001) with the greatest improvement in Data Collection and Analysis (+ 34.65%), and in self-efficacy scores across all domains (*p* <  0.001). 166/307 (54.1%) participants responded to the 6 months post-RWs follow-up survey. Compared to pre-RWs, Research involvement increased from 40.4 to 62.8% (*p* <  0.001) while proportion of participants with peer-reviewed publications increased from 8.4 to 15.8% (*p* = 0.043).

**Conclusion:**

Virtual RWs allow for a wide outreach while effectively improving research-related knowledge and skills, with minimal associated costs. In lower-middle-income countries, virtual RWs are a creative and cost-effective use of web-based technologies to facilitate medical students to contribute to the local and global healthcare research community.

## Introduction

Health research is crucial for practicing evidence-based medicine [1]. Physician-investigators play an important role in the research community, as they translate the progress in basic sciences to a clinical setting. However, though South Asia produces many health professionals across various fields and allows several health-related research opportunities, there is yet a serious paucity of scientific publication. South Asia contributes only 1.2% of all health science research within the Institute for Scientific Information database from 1992 to 2001 [2]. Pakistan contributes less than 0.1% of all research publications globally [3], and spends only 0.3% of its gross domestic product (GDP) on research and development [4].

Moreover, only few medical students from low-middle-income countries (LMICs) like Pakistan are involved in research [2]. The participation of medical students in research is hindered by the lack of training, dedicated and seasoned mentors, and lack of research opportunities [2, 5]. Unfortunately, research mentoring and career counselling has historically been a voluntary activity in most institutions. This restricts young researchers from gaining professional progress or forming precise career goals [6].

Early exposure to research can be beneficial for medical students in improving skills such as searching and critically appraising the medical literature, independent learning, and manuscript writing [2]. Moreover, students with research experience during medical school have higher chances of careers in academic medicine [2, 7]. While previous studies have demonstrated the effectiveness of research workshops covering solitary aspects of research [8, 9] among medical students [7], currently there are very few studies assessing the impact of a comprehensive research course, particularly in the setting of an LMIC like Pakistan. In spite of efforts made by Higher Education Commission (HEC) Pakistan, the research systems are not performing as desired and the students of Pakistan’s medical colleges have not shown much involvement in research [2] [4, 10]. Initiatives such as research workshops (RWs) need to be conducted to improve the health research culture in Pakistan. LMICs such as Pakistan have low budget allocated on healthcare research due to the struggling economy. Hence, it is important to consider the financial constraints and have workshops that are low-cost, accessible and provide quality education regarding research [11].

The COVID-19 pandemic has created a health care crisis around the world and has presented a spectrum of challenges, including a profound effect on the delivery of education. Institutions are being forced to transition to online and remote education [12]. Moreover, there has been focus on virtual peer teaching due to its various potential advantages [13]. Peer teaching is considered as an efficient method of teaching, providing a better learning experience, and leading to improved performance [14, 15]. A study by Steinert et al., albeit amongst medical faculty members, reported that integration of peer support groups for manuscript writing have led to better research outcomes [16]. Amongst medical students too, peer-teaching has been shown to be a highly effective educational strategy, producing outcomes comparable to [17], or, in some respects, even superior [18] to conventional faculty-led teaching. Virtual peer teaching can be part of the solution to challenges in medical education during the pandemic. Therefore, there is a dire need to creatively use web-based technologies and introduce innovative virtual-learning opportunities for students. In this study, we aim to assess the effectiveness of peer-taught virtual research workshops in improving the research-related knowledge and skills of medical students in Pakistan. In addition, we compare the costs associated with the virtual research workshops to those associated with in-person research workshops.

## Methods

### Study design and setting

A quasi-experimental study was conducted by the Society for Promoting Innovation in Education (SPIE), in collaboration with the International Federation of Medical Students’ Associations (IFMSA) – Pakistan, Department of Biological and Biomedical Sciences (BBS) and the Center for Innovation in Medical Education (CIME), at the Aga Khan University (AKU), Pakistan. Five research workshops (RWs) were conducted between 20^th^ and 26^th^ July 2020 over the online video conferencing platform Zoom, due to feasibility during the ongoing COVID-19 pandemic. The technical analysts’ team at CIME, AKU provided the technical support and service required for the RWs The peer-taught research workshops (RWs) were carried out after prior approval from the institutional ethics review committee (Reference Number: 2020–1362-10,219).

### Setting in context: medical School in Pakistan and SPIE

In Pakistan, medical school is a 5-year undergraduate program (Bachelor of Medicine, Bachelor of Surgery; MBBS) that is entered directly after high-school i.e., enrolment in a pre-medical college program is not a pre-requisite. In medical schools in Pakistan, the first 2 years (Years 1 and 2) are pre-clinical years, where students are taught the basic sciences. Following that, Years 3–5 are clinical years, during which students complete clinical rotations. Throughout medical school, a variety of teaching pedagogies are employed, including conventional didactic lectures, problem-based learning, and team-based learning. The degree to which these different pedagogies are used amongst medical schools.

The Society for Promoting Innovation in Education (SPIE) is a student-run, non-profit, organization, founded in 2017 by the medical students at AKU. SPIE aims to promote culture of innovative learning and revolutionize educational practices. SPIE has five wings, including Research and Development Wing (R&D Wing). The RWs were organized by SPIE’s R&D Wing, in collaboration with International Federation of Medical Students Associations (IFMSA), Pakistan. Through this collaboration, SPIE was able to expand its outreach to medical colleges across the country, both public- and private-sector, hence improving national generalizability. Additionally, SPIE has previously published a protocol [19] for a similar peer-taught virtual RW series for surgical trainees, and this may be referred to for further details on the methodology for a peer-taught online RW series.

### Participant recruitment and enrolment

The minimum required sample size calculated using University of California San Francisco Calculator [20] was 161, with alpha 0.05, beta 0.20, effect size 0.500, and standard deviation (SD) of the post-over-pre change of 2.25. The SD was calculated using the following formula from a previously published study with a similar study design [21], with standard error of mean (SEM) 1 and 2 computed from SD on the pre-test (16.11%) and post-test (12.39%), respectively.

The recruitment for participants of the workshops was carried out via a Google Form which was disseminated on SPIE’s social media platforms including Facebook, Twitter and WhatsApp. The target population of the RW participants consisted of medical students from all over Pakistan. The inclusion criteria consisted of students aged 18 years or above currently enrolled in an MBBS program in a Medical College of Pakistan. The first 400 registrations meeting the inclusion criteria were selected and enrolled (convenience sampling). Despite the minimum required sample size being 161 only, 400 participants were enrolled to maximize benefit of the RW series at no additional cost or compromise in the quality of the RWs. A confirmation email, along with a consent form (see Ethical Considerations) was sent to these 400 participants. In order to assess the outreach of our recruitment process, a region-wise percentage of included medical colleges was calculated across the following administrative regions in Pakistan (*n* = 117) [22]: Sindh (*n* = 29), Punjab (*n* = 62), Baluchistan (n = 2), Khyber Pakhtunkhwa (*n* = 20), and Azad Jammu and Kashmir (*n* = 4).

Exclusion criteria consisted of an incomplete attendance which referred to having missed the pre-RW or post-RW quiz of more than 1/5 RWs. Adequate attendance was having completed a minimum of 4 out of 5 pre-and post-RW quizzes. In addition, participants were also required to fill a post-RW series feedback form (see Data Collection) as part of the criteria for workshop completion. Upon completion of the RWs, the participants received a certificate of participation as an inducement if they fulfilled the attendance criteria of the RWs.

### Research workshop content and curriculum development

**Fig. 1:**
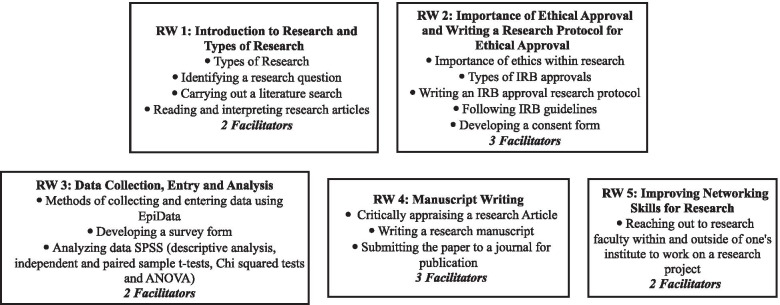
Schema of Research Skills taught during five Research Workshops

The content and structure of the RWs were developed in association with research faculty at AKU. Figure 1 shows the content of all 5 workshops and the number of facilitators required for each workshop. Briefly, the skills taught in the 5 RWs included initiating research, manuscript writing, considering ethics in research, data mining and statistical analysis, and networking skills. Each individual workshop was completed within 3 h on a single day (including the pre- and post-RW quizzes and self-efficacy forms), for a maximum of 15 h over a total of 5 days. Multiple teaching methodologies were implemented, including didactic lectures and presentations, interactive discussions, formative quizzes. Questions from the participants were welcome at any time during the RWs.

### Facilitator training and mock workshops

A total of 12 facilitators were recruited and trained to conduct the 5 RWs. In order to maintain a peer-education model, facilitators were chosen from the “Student Research Mentors” team of SPIE. The student research mentors are medical students at AKU with prior research experience conducting and mentoring fellow medical students on at least one research project previously, as part of SPIE’s Student Research Mentorship Program [23]. The student research mentors volunteered to facilitate the current RW series. These facilitators were from Years 1–5 of MBBS. Training of facilitators took place in the form of five small-group sessions, whereby the facilitators (i.e., student research mentors from SPIE) were trained by faculty from the Department of BBS. Apart from subject knowledge and skills pertaining to research, facilitators were also acquainted with teaching styles (expert, authority, demonstrator, facilitator, and delegator) [24] and methodologies (didactic presentations, interactive activities, formative quizzes, team-based activities, problem-based learning, and learner-learner and facilitator-learner feedback). Mock sessions led by the facilitators were conducted for student members of SPIE to ensure any issues regarding content, level of interactivity, hardware, software, time management and achievement of learning objectives could be identified. In these mock sessions, the pre- and post-RW quizzes and self-efficacy forms, and feedback forms were piloted. While the final content, structure, and style of the RW’s was vetted by the faculty from the Department of BBS, strict standardization of teaching styles and methodologies was not attempted as this may have restricted the organic nature of the peer-teaching relationship.

### Data collection

The following Google Forms were filled by the participants of the RWs series**:**

- *Participant Sign-Up Form*: Student demographics were collected in the Google Form which was used for registration. These included age, gender, and year of study in medical school (years 1 and 2: pre-clinical; years 3–5: clinical). In addition, students were asked if they had ever been involved in conducting research before, and if they had ever attended a RW before. Though all 400 participants would have filled the sign-up form, only those meeting minimum criteria for completion were included in the final analysis.
- *Pre-and Post-RW Quizzes*: Each participant was required to take a quiz before and after attending each RW, to evaluate the effectiveness of the respective RWs. Both the pre- and the post-quiz for each RW were identical, and tested participants’ research-related knowledge and application of research-skills. Out of the 400 enrolled participants, only those attending each individual RW were allowed to attempt the quiz for that specific RW. Students were only required to attempt the quizzes in order to fulfil attendance criteria, with scores on pre- and post-RW quizzes not impacting completion of the RW series. The pre- and post-RW quizzes were piloted during the aforementioned mock sessions, and ambiguous questions were amended. The Cronbach’s alpha for the pre- and post-RW quizzes were 0.61 and 0.78, respectively, indicating acceptable to good reliability of the quizzes.
- *Pre- and Post- Self-Efficacy Forms*: Participants were required to rate their confidence in 16 pre-defined objectives (detailed later in Table 3) on an integer scale of 1–10, where 1 represented low confidence and 10 high confidence. These self-efficacy forms were filled by attendees before and after the RW series, with their self-administered nature precluding observer bias, to evaluate self-perception of learning. The self-efficacy forms were developed by SPIE and piloted in the mock sessions mentioned above, where no changes were deemed necessary. The Cronbach’s alpha for the pre- and post-RW self-efficacy forms were 0.96 and 0.98, respectively, indicating excellent reliability of the self-efficacy forms. Though all 400 students were emailed self-efficacy forms to complete, only those who had met completion criteria for the RW series were included in analysis.
- *Feedback Forms*: Following completion of the series there was a feedback form which the participants were asked to fill. All the data collected was kept anonymous and no personal identifiers were asked. The feedback will help the organizers improve future workshops and gauge the overall reception and digestibility of the RW series. 400 enrolled participants were expected to fill out the form, as this was a criteria for RW series completion. The feedback forms were piloted during the mock sessions, and demonstrated good internal consistency with a Cronbach’s alpha of 0.897.
- *6-months post-RW series Follow-Up Survey*: A follow-up survey was emailed to all the enrolled participants. The survey was conducted to assess the efficacy of workshops based on tangible long-term improvement in research participation and output. Only participants who had met the minimum criteria for completion, and had received a Certificate of Completion, were emailed the 6-months post-RW series follow-up survey.

For cost comparisons, an audit of SPIE’s financial expenditures associated with previous in-person research workshops which took place in January 2020 was conducted to compare costs with those associated with the current virtual RWs.

### Ethical considerations

Informed consent was obtained via email, whereby the 400 selected participants were emailed a consent form and required to provide their informed consent for participation in the quasi-experimental study. The consent form explained the scope of the RW series, the degree of attendees’ involvement, and the use of attendees’ data for research. Moreover, attendee’s right to withdraw at any point during the RW series was explained. There were no risks or costs for attendees, and the only inducement was a Certificate of Completion.

De-identification and confidentiality of participants’ data was ensured. A unique identifier number (UIN) was provided to all participants in the initial enrolment email, and all data collected subsequently was done so using the UIN. Only two research team members, RDU and MAB, assigned and emailed the UINs to selected participants, and they were also responsible for tallying attendance in order to provide Certificates of Completion. However, RDU and MAB did not partake in data handling, so as to maintain participants’ anonymity.

### Data analysis

Analysis was performed using IBM SPSS for Windows, version 23.0 (IBM Corp., Armonk, N.Y., USA). Categorical data was described as frequencies (n) and percentages (%). Continuous data was expressed as mean and standard deviation. Paired t-tests were used to compare mean pre-and post-research workshop quiz scores and self-efficacy scores. Categorical data was compared using Chi-squared tests, while continuous data was compared using independent sample t-tests. McNemar’s test was used to compare paired responses to dichotomous variables in the 6-month follow-up surveys (e.g., paired subject responses to “Did you have any research involvement before you participated in the RWs? Yes/No” vs. “Are you involved in any research activity currently, 6-months post RW series? Yes/No”). A *p*-value < 0.05 was regarded as significant for all analyses.

## Results

Out of the 400 medical students enrolled from 36 medical colleges in Pakistan, a total of 307 (76.8%) medical students met the minimum completion requirements. Amongst the 36 medical colleges included, 21 (58.3%) were public-owned while the rest were private-owned. The province−/region-wise distribution of the 36 medical colleges were as follows: 17 (/62; 27.4%) from Punjab, 14 (/29; 48.3%) from Sindh, 3 (/20; 15%) from Khyber Pakhtunkhwa, 1 (/2; 50%) from Baluchistan, and 1 (/4; 25%) from Azad Jammu and Kashmir. The mean age of the students was 21.37 ± 1.91 years. Across the 5 RWs, the maximum attendance was seen in RW1 (352/400; 88%) and RW3 (341/400; 85.3), while lower attendance was seen in RW2 (312/400; 78%), RW4 (301/400; 75.3%), and RW5 (290/400; 72.5%). 56.4% of the participants belonged to the pre-clinical years, with a year-wise breakdown as follows: 80/307 (26.1%) from Year 1, 93/307 (30.3%) from Year 2, 26/307 (8.5%) from Year 3, 64/307 (20.8%) from Year 4, and 44/307 (14.3%) from Year 5. Most students (60.9%) did not have any prior research experience and the majority (59.0%) had not attended any Research workshop previously. The demographics are summarized in Table 1.

**Table 1:** Demographics and Baseline Characteristics

Variable	Overall (*N* = 307)
**Age**	21.37 ± 1.91
**Gender**	
**Male**	117 (38.1)
**Female**	190 (61.9)
**Year of Medical School**	
**Pre-Clinical**	173 (56.4)
**Clinical**	134 (43.6)
**Have you ever been involved in conducting research before?**	
**Yes**	120 (39.1)
**No**	187 (60.9)
**Have you ever attended a RW before?**	
**Yes**	181 (59.0)
**No**	126 (41.0)

The students demonstrated significant improvement in scores from pre-workshop quiz to post-workshop quiz (p <  0.001). The greatest improvement in scores was shown in RW 3 (Data Collection, Entry and Analysis) with mean difference of 34.65% ± 24.49%. The results for pre-and post-workshop quizzes are summarized in Table 2.

**Table 2:** Pre- and Post-workshop Quiz Scores

Research Workshop	Pre-RW Quiz Score (%) (Mean ± SD)	Post-RW Quiz Score (%) (Mean ± SD)	Difference in Post- and Pre- Scores (%) Mean ± SD	P-Value
**RW 1**	47.92 ± 20.55	78.54 ± 20.69	30.62 ± 21.20	< 0.001
**RW 2**	55.74 ± 17.02	79.38 ± 16.99	23.63 ± 18.37	< 0.001
**RW 3**	26.42 ± 12.76	61.07 ± 24.94	34.65 ± 24.49	< 0.001
**RW 4**	51.34 ± 17.97	62.68 ± 20.38	11.33 ± 18.27	< 0.001
**RW 5**	54.56 ± 21.54	72.04 ± 23.91	17.49 ± 21.24	< 0.001

The difference in the mean improvement of pre-workshop and post-workshop quiz for RW 5 (Improving Networking Skills for Research) was significantly higher in the pre-clinical students compared to clinical students (19.89% ± 21.48% vs. 14.38% ± 20.59%; *p*-value = 0.024). In addition, the difference in the mean improvement of pre-workshop and post-workshop quiz for RW 5 (Improving Networking Skills for Research) was significantly higher among the participants who had conducted research before compared to the participants who had not conducted research previously (14.55% ± 18.95% vs. 9.27% ± 17.57%; *p*-value = 0.013).

The pre- and post-workshop analysis revealed a significant improvement in all the 16 self-efficacy scores grouped into 5 categories (*p* <  0.001). The greatest improvement in self-efficacy scores were for “Understanding an ANOVA test” (4.56 ± 3.33) followed closely by “Understanding the types of T-tests” (4.35 ± 3.33). The results are summarized in Table 3.

**Table 3:** Pre-and Post-RWs Self Efficacy Scores

	Pre-RW (/10)	Post-RW (/10)	Post-Pre (/10)	P-Value
Research Skills	(Mean ± SD)	(Mean ± SD)	(Mean ± SD)	
**Initiating Research (RW1)**				
**Identify a good research question**	3.88 ± 2.40	7.06 ± 2.42	3.18 ± 2.20	< 0.001
**Conduct a database literature search**	4.13 ± 2.67	7.15 ± 2.49	3.02 ± 2.33	< 0.001
**Manuscript Writing (RW1 and RW4)**				
**Critically appraise research articles**	3.49 ± 2.66	6.65 ± 2.51	3.16 ± 2.48	< 0.001
**Writing a research manuscript**	3.57 ± 2.73	6.80 ± 2.52	3.22 ± 2.49	< 0.001
**Considering Ethics in Research (RW2)**				
**Avoid plagiarism in your manuscript**	4.91 ± 3.03	7.17 ± 2.70	2.26 ± 2.39	< 0.001
**Take ethical issues into consideration**	5.15 ± 3.08	7.72 ± 2.57	2.57 ± 2.59	< 0.001
**Data Mining and Statistical Analysis (RW3)**				
**Understand data and types of variables**	3.74 ± 2.83	7.33 ± 2.62	3.59 ± 2.70	< 0.001
**Designing a research survey**	4.12 ± 2.79	7.11 ± 2.49	3.00 ± 2.43	< 0.001
**Designing a survey-based methodology**	3.90 ± 2.80	6.90 ± 2.50	3.00 ± 2.52	< 0.001
**Understand descriptive analysis**	4.39 ± 2.96	7.41 ± 2.57	3.02 ± 2.62	< 0.001
**Understand the types of T-tests**	3.04 ± 2.93	7.39 ± 2.71	4.35 ± 3.33	< 0.001
**Understand Chi-Square tests**	3.35 ± 3.07	7.29 ± 2.71	3.94 ± 3.17	< 0.001
**Understanding an ANOVA test**	2.49 ± 2.82	7.05 ± 2.73	4.56 ± 3.33	< 0.001
**Networking Skills (RW5)**				
**Writing a professional email**	4.49 ± 2.95	7.46 ± 2.58	2.97 ± 2.56	< 0.001
**Networking ability**	4.19 ± 2.85	7.12 ± 2.62	2.93 ± 2.48	< 0.001
**Choosing the right research mentor**	3.81 ± 2.85	7.01 ± 2.62	3.20 ± 2.59	< 0.001

In comparison to participants with no prior research experience, the difference in the mean improvement of self-efficacy scores in “Understand the types of T-tests” (3.95 ± 3.05 vs. 4.98 ± 3.40; *p* = 0.006), “Understand Chi-Square tests” (3.58 ± 2.90 vs. 4.49 ± 3.28; *p* = 0.011), Compared to participants without prior research experience, participants with prior experience demonstrated a significantly greater improvement in “Understanding an ANOVA test” (4.20 ± 3.10 vs. 5.11 ± 3.35; *p* = 0.015).

The feedback given by the participants showed that the RWs were well received. More than 90% of the participants believed that the overall organization of the online RWs, quality of presentation and discussion, and relevance to current level of expertise, was either “Excellent/Good”. All the RWs were rated at least satisfactory and above. The feedback is summarized in Table 4.

**Table 4:** Feedback of RW Series

Aspect of RW Series ^**a**^	Overall (N = 307)
**Overall Organization of Online RWs**	
**Excellent/Good**	285 (92.8)
**Satisfactory**	22 (7.2)
**Time Allotment**	
**Excellent/Good**	252 (82.1)
**Satisfactory**	46 (15.0)
**Quality of Presentation**	
**Excellent/Good**	277 (90.2)
**Satisfactory**	29 (9.4)
**Effectiveness of Peer Teachers**	
**Excellent/Good**	276 (89.9)
**Satisfactory**	28 (9.1)
**Quality of Discussion**	
**Excellent/Good**	280 (91.2)
**Satisfactory**	24 (7.8)
**Interestingness**	
**Excellent/Good**	261 (85.0)
**Satisfactory**	43 (14.0)
**Quality of Course Materials**	
**Excellent/Good**	276 (89.9)
**Satisfactory**	28 (9.1)
**Relevance to Current Level of Expertise**	
**Excellent/Good**	278 (90.6)
**Satisfactory**	27 (8.8)
**Relevance to Audience**	
**Excellent/Good**	270 (87.9)
**Satisfactory**	36 (11.7)
**Appropriateness of Level of Difficulty**	
**Excellent/Good**	249 (81.1)
**Satisfactory**	54 (17.6)

^a^ Responses coded Poor/Very Poor not shown – not more than 3% of responses to any question

In comparison to the pre-clinical year students (79.2%), clinical years students believed that the time allotment was “Excellent/Good” (85.8%; *p* <  0.001).

### 6-month post-RW series follow-up

Out of the 307 medical students who participated in the RWs, 166 participants responded to the 6-month follow-up survey (response rate: 54.1%). The majority (*n* = 110; 66.3%) reported that participation in the RWs had enhanced their knowledge and skills to conduct research independently “Very Significantly/Significantly”, while the remainder reported that it had helped them “Moderately”. 62.8% students reported involvement in research projects during the 6-months after attending the RWs, as compared to their involvement before attending the RWs (40.4%; *p* <  0.001). In comparison to 14 (8.4%) participants with peer-reviewed publications before the RWs, 26 (15.8%) students reported having peer-reviewed publications 6 months after RWs (*p* = 0.043). The results of the follow-up survey are shown in Table 5.

**Table 5:** Follow-Up Data from 6-Months Post-RWs

Variable	Follow-Up (***N*** = 166)		***P***-Value
	Before RWs n (%)	After RWs n (%)	
**Research Involvement**	67 (40.4)	103 (62.8)	< 0.001
**Number of Research Projects Involved In**			< 0.001
**0**	99 (59.6)	64 (38.5)	
**1–2**	53 (31.9)	57 (44.2)	
**3–4**	11 (6.6)	24 (18.6)	
**> 5**	3 (1.9)	21 (12.7)	
**Peer-Reviewed Publications**	14 (8.4)	26 (15.8)	0.043

### Costs

For the in-person RWs conducted by SPIE in January 2020, the total cost amounted to approximately USD 2235.36. The virtual RWs were conducted on Zoom. The technical analysts’ team at CIME, AKU provided the technical support and service required for the RW series. The Zoom package, already purchased by CIME, and technical support staff, was provided by CIME to SPIE for free of cost. There was no cost invested by SPIE. However, to present a holistic comparison of costs, support staff and Zoom costs are considered as expenditures for the virtual RWs. Even so, the virtual RWs catered to more than three times as many students as the in-person RWs at about 5% the total cost (Table 6).

**Table 6:** Comparison of In-Person vs. Virtual RWs

Variables	Cost					
	In-Person RWs				Virtual RWs	
	One Participant		100 Participants		307 Participants	
	PKR	USD	PKR	USD	PKR	USD
**Breakfast**	800	5	80,000	499.69	0	0
**Lunch**	1000	6.25	100,000	624.61	0	0
**Refreshments**	300	1.87	30,000	187.38	0	0
**Certificates**	50	0.31	5000	31.23	0	0
**Fixed Venue Cost**	144,000	899.44	144,000	899.44	0	0
**Support Staff**	2600	14.3	2600	14.3	2600	14.3
**Zoom**	0	0	0	0	15,882	99.0
**Total**	146,150	912.87	359,000	2235.36	18,482	113.3

## Discussion

The rapidly evolving crisis, the COVID-19 pandemic, has led to several challenges with respect to education. It has led to development of various forms of educational systems including peer teaching and utilization of virtual platform to meet the educational needs [13]. The usefulness of peer teaching and online learning in health sciences education is well known [13]. We conducted a study to determine the impact of virtual peer teaching and effectiveness of low-cost research workshops in the setting of an LMIC like Pakistan. The results of our study highlighted that the virtual peer-taught workshops were effective as there was a significant improvement in the pre-and post-quizzes scores of all five RWs. The improvement in self-efficacy scores across all 16 domains also reflected that the RWs were effective. In addition, the RWs were received well by the participants, as demonstrated in their evaluations where they reported good levels of satisfaction particularly regarding peer-teaching and online organization of RWs. The results of follow-up survey showed there was a rise in research involvement and peer-reviewed publications. Lastly, the outreach of online RWs was seen to be quite considerable, as students from 36/117 medical colleges in Pakistan were able to take part.

Research training is an important aspect of medical education to practice evidence-based medicine in healthcare [7]. Perhaps, institutions should take initiatives such as research training workshops as part of the medical curriculum as a sustainable and highly effective approach in improving medical students research training and skills. The findings of our study suggest medical students actively learn from RWs and grasp basic key research skills that can be used to conduct research. The pre- and post- workshop analysis revealed a significant improvement in scores of quizzes of all five RWs and all the 16 self-efficacy scores. Amongst the 16 objectives measured by the self-efficacy questions, the greatest improvement in self-efficacy was seen in objectives covered under the Data Mining and Statistical Analysis RW. In addition, participants with prior research experience showed higher mean improvement in self-efficacy scores of statistical analysis domains. The results of our follow-up survey showed that the RWs have been effective in improving the research-related knowledge and skills of our participants, while also providing a degree of long-term validation to the improvements we observed on the pre- and post-RW quizzes and self-efficacy forms. From 77 participants who responded to the follow-up survey, only 7.8% of students were involved in > 2 projects before RWs, compared to 14.3% of students involved in > 2 projects after the RWs. In addition, 15.8% of students had at least one peer-reviewed publication 6 months after the RW series, compared to only 8.4% before. It is possible that these were projects initiated or in process before the RW series, given that it is unlikely for projects to be completed from scratch to publication within 6 months. However, it is also possible that participants were able to use their newly learned research and networking skills to get involved in research projects already underway with faculty (almost two-thirds of students were involved in research after the RW series, compared to less than half before). Thus, the direct impact of the RW series on participants’ actual research involvement and success cannot be discounted, especially as two-thirds of participants reported that the RW series had helped them very significantly/significantly to conduct research independently. The results of study by Antonou et al. revealed that students had shown improvement in self efficacy scores as a result of attending research workshops [25]. More than 90% of the participants believed that the overall organization, quality of presentation and discussion, and relevance to current level of discussion was either “Excellent/Good”. Similarly, a study from Egypt has shown the positive outcomes and effectiveness of online medical research skills workshops [26]. Thus, there is a need to conduct RWs for medical students to learn to properly conduct research and highlight the importance of research in their profession [5, 27].

In our study, students demonstrated significant improvement in scores from pre-workshop quiz to post-workshop quiz and all the 16 self-efficacy scores pertinent to the basic research skills. Similarly, a study conducted by Morales-Pérez et al. reported significant improvement in the pre-and post- test scores of the participants after attending an online course [28]. Moreover, the outcomes of a study conducted by Soffer et al. showed that students scored higher in online courses than in face to face courses [29]. More than 90% of the participants believed that the overall organization of virtual RWs was either “Excellent/Good”. A similar pattern of good feedback by the students is reflected in previous studies about online courses [30]. Online modalities have led to high student satisfaction as learning is more student-centered [31, 32].

Virtual mode of learning is more convenient, saves resources, quick and efficient, and gives students the opportunity to become independent learners [32]. Our RW series was able to accommodate 400 students from 36 different medical colleges in Pakistan, which bears testimony to the far-reaching accessibility of online learning. In addition, online mode of learning leads to significant cost savings, especially considering the financial constraints of LMICs such as Pakistan [33, 34]. Our results demonstrate the cost-effectiveness and scalability of virtual RWs compared to their in-person counterparts, as we were able to successfully teach more than three times the number of students with just 5% of the cost. On the other hand, virtual learning comes with its own limitations such as hardware and software issues, internet connectivity issues and other technical problems [30]. Lack of availability of internet in remote areas of Pakistan or unscheduled power cuts during the virtual RWs represent possible challenges. The findings of our study reflect the positive perception of the virtual workshops. Thus, virtual medium is a cheap and highly effective approach to tackle the problem of medical research in Pakistan, especially in COVID-19 pandemic times.

Peer teaching is an effective method of education delivery that promotes a learning environment among peers [35]. The challenges imposed by the rapidly evolving crisis, COVID-19 pandemic, has led to limited access to conventional teaching style and more emphasis on peer-taught session [30, 35]. The results of our study show that peer taught workshops were effective as students showed significant improvement in scores from pre-workshop quiz to post-workshop quiz. Moreover, the feedback given by the participants reflected that the workshop was well received, and participants were satisfied. In addition, 89.9% of the participants believed that the effectiveness of peer teachers was either “Excellent/Good” as peer teachers are more approachable. A study be Dehghani et al. revealed that peer teaching is an effective mode of learning as there was significant improvement in test scores and participant satisfaction was approximately 88% [15]. Furthermore, previous studies have shown peer teaching has led to better results in comparison to faculty instructors [13, 36]. Peer taught workshops are an example of sustainable phenomena as peer teachers can mentor and support peer learners to further teach more novice students.

This study had a few limitations. Though the pre-post design of this quasi-experimental study ensured a high internal validity, a degree of external validity is sacrificed due to the nature of the study design. Also, performance on the pre- and post-quizzes could be affected by external influences other than the RWs themselves, especially if participants attempted to supplement their learning at the RW series by consulting other resources. Additionally, there is no absolute way of knowing to what extent the heartening results at the 6-month follow-up in our study are attributable purely to the RW series.

The pre-RW quizzes may have sensitized participants and led to bias in participants’ responses when answering the post-RW quizzes. In addition, there was no way invigilate students while they responded to the pre- and post-RW quizzes, and results may be biased by the honesty of participants’ quiz-taking behavior. Moreover, as scores on the quizzes did not impact workshop completion, participants may have not filled it with their complete attention. Furthermore, although self-efficacy has been used previously to evaluate learners’ perceptions of their own improvement, this rating system is undeniably subjective and does not in itself prove objective increase in expertise. Lastly, the lack of availability of internet facility in remote areas or unscheduled power cuts during the sessions were another challenge in the setting of an LMIC like Pakistan.

## Conclusion

The present study conducted by SPIE demonstrates the utility of virtual research workshops in improving the research-related knowledge and skills among medical students. The virtual nature of research workshops allows for a much wider outreach than conventional in-person research seminars. In addition, low-cost virtual research workshops are a creative use of web-based technologies to facilitate medical students to contribute to the local and global healthcare research community. Therefore, workshops conducted by SPIE can be easily replicated on a much larger scale to promote research culture in a cost-effective way.

## Data Availability

All data generated or analysed during this study are included in this published article (and its supplementary information file). The data that support the findings of this study are available from the corresponding author upon reasonable request.

## Abbreviations

SPIE: Society for Promoting Innovation in Education
AKU: Aga Khan University
RWs: Research Workshops
LMICs: Low-Middle-Income Countries
HEC: Higher Education Commission
IFMSA: International Federation of Medical Students’ Associations
CIME: Center for Innovation in Medical Education
